# Editorial: Coronavirus Disease (COVID-19): Psychological Reactions to the Pandemic

**DOI:** 10.3389/fpsyg.2021.745941

**Published:** 2021-08-30

**Authors:** Joanna Sokolowska, Peter Ayton, Eduard Brandstätter

**Affiliations:** ^1^Faculty of Psychology, University of Social Sciences and Humanities, Warsaw, Poland; ^2^Centre for Decision Research, University of Leeds, Leeds, United Kingdom; ^3^Department of Economic Psychology, Johannes Kepler University of Linz, Linz, Austria

**Keywords:** COVID-19, risk, fear, pandemic, mental health, social behavior, emotion

*Geneva, Switzerland, January 30, 2020*. It is no exaggeration to claim that this day marked the official beginning of a new experience—or even an ordeal—for billions of people around the globe. On this day, Adhanom Ghebreyesus, the director-general of the World Health Organization stepped in front of the microphones and officially declared “a Public Health Emergency of International Concern (PHEIC) over the global outbreak of a novel Coronavirus,” which constitutes “an extraordinary event which is determined to constitute a public health risk to other States through the international spread of disease and to potentially require a coordinated international response” (WHO, 2019). nothing less than WHO's highest alert level. Given the novelty of this situation, most citizens and politicians did not grasp what was just happening despite the declaration's explosive nature. Tourists and locals, for instance, continued partying in Alpine ski resorts, politicians tried to cope with the problem by betting on herd immunity through natural infection, and world leaders reacted by simply denying the severity of the new threat. Still others, seeking to play down the crisis, drew parallels to the 2009 swine flu pandemic which also triggered a WHO PHEIC, resulting in significant reaction in the media, but which, studies suggest, resulted in no greater number of deaths than the numbers dying annually of seasonal flu (Belongia et al., 2010).

In retrospect one reason why reactions to the WHO announcement may have been subdued is because the term PHEIC isn't as impactful, as emotive—or even as recognizable—a term, as, say, “pandemic” or “emergency.” Apparently, researchers and health officials advising WHO deliberately chose this term, rather than a more impactful one, in part because they wanted to avoid panic while encouraging world leaders to act according to WHO advice in order to contain a threat (Maxmen, 2021). In any event what happened next produced a global impact on human history. A virus the size of a 10 thousandth of a millimeter forced billions of people to drastically change their life routines, ranging from the private to the public.

The first reactions from the scientific community were diversified. Early in the pandemic, several eminent behavioral scientists claimed in commentaries that the psychological evidence from research on behavior under risk indicated that people would overreact to the risks posed by the pandemic and panic. These claims made in various prominent—but not peer-reviewed—publications came under strong criticism, also not from a peer-reviewed source (Richie, 2020), for the paucity and inappropriateness of the evidence on which they were based and appear to have been quietly dropped by their proposers. Here one may see the curse of hindsight: in an emergency situation where data are sparse but rapid action may pay dividends (and the beginning of the pandemic was clearly such an emergency situation) decision makers and analysts look for cases from their past experience that resemble the current one. If they find a clear match, they can carry out the most typical course of action. By that method, people can successfully make extremely rapid decisions (Klein, 2008). Clearly, this is not guaranteed to provide optimal solutions, but is the best one can do in such a situation, and, on average, will be better than doing nothing.

In spite of arguments about some behavioral scientists' assessments very early in the development of the pandemic there were some other valuable contributions from behavioral science. At least one behavioral scientist warned that a more likely public response than over-reaction and panic, that itself posed a real danger, was the exact opposite reaction: complacency inspired by underestimation of the threat (Carey, 2020). To account for the diverse responses to the pandemic Chater (2020) aptly pointed out the well documented tendency of people to impose a single interpretation on ambiguous situations without entertaining alternatives which, while often serving us well, can lead to disastrous outcomes. Moreover, an open letter signed by over 600 behavioral scientists challenged the UK government's apparently baseless presumption that a lockdown should not be introduced early as the population would suffer “behavioral fatigue.” This event and the curious, and indeed dubious, invocation of the concept of behavioral fatigue was subsequently described and analyzed by a paper published in this Research Topic (Harvey).

There are perhaps lessons in these events that might be drawn for behavioral scientists attempting to advise on future human crises where it seems that there is an established evidence base that might be readily applied to a novel problem for which, understandably there is no direct data. Advice and recommendations putatively drawn from the relevant science should, in advance of being widely disseminated, be tested in contexts as similar as possible to those where they are being applied. Our Research Topic does serve this objective by reporting considerable amounts of empirical data directly arising from the pandemic.

Putting these and other irrationalities aside, many others, including politicians, citizens, and scientists reacted responsibly and quickly by searching for constructive solutions. Virologists started searching for a new vaccine, economists investigated the financial effects of the lockdown, and psychologists tried to gain a better understanding of the psychological reactions to the pandemic. Both the huge range of areas where behavioral science might make a contribution (Van Bavel et al., 2020) and the need for caution in generalizing from pre-pandemic phenomena (IJzerman et al., 2020) were prominently signaled to the research community.

One of the many upshots of this quest for a better grasp of the cognitive, emotional, and behavioral reactions triggered by the pandemic was our decision to edit a special Research Topic as quickly as possible. This effort was further motivated by our desire to find solutions that would help people to cope with the many adversities arising from the pandemic, such as lockdown, loneliness, stress, or economic hardship: “How do people cope with risks and uncertainties related to the pandemic?,” “What are the psychological influences on economic behavior?,” or “What are the psychological processes accompanying pandemic judgment and decision making among experts and the lay population?” are just a few of the topics that we invited contributors to address in our call for papers published online on April 8th 2020.

Considering the very short period from the onset of the pandemic, we had some initial concerns about not attracting enough contributions, but the submission of more than 200 abstracts and manuscripts exceeded our wildest expectations. The submitted topics ranged from stress to coping and from perception to culture. To get a clearer picture, the word cloud shown in Figure 1, which was derived from the key words of the published articles in this Research Topic, depicts the frequency of each key word by its size.

**Figure 1:**
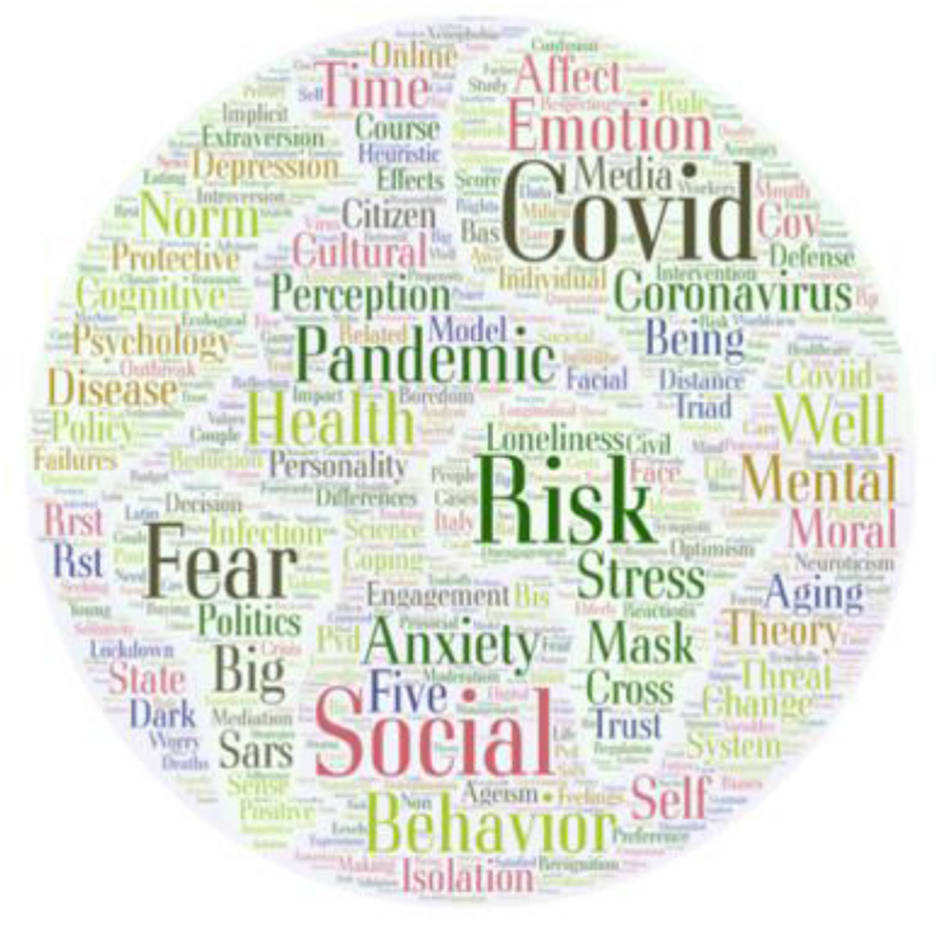
The word cloud derived from the key words of the published articles in this Research Topic.

Figure 1 shows that the 257 authors of this Research Topic most often investigated questions related to risk and affect, including the painful emotions of fear and anxiety. Other contributors invested their energy to find answers to questions concerning people's health worries, stress responses, or coping strategies. Yet other contributors were attracted by the socio-emotional tensions caused by mask-wearing, and still others researched and found new insights into the effects of social isolation on well-being. Their efforts yielded findings that, hopefully, will contribute to the efforts to help alleviate the suffering caused by Covid-19 as well as potential future pandemics.

Having said that, some topics in our call for papers received much less attention. Examples include judgment and decision making, economic hardship, or issues related to politics. One reason for this underrepresentation might simply be the smaller number of researchers working in these areas. Another interpretation might be that the more popular topics in this e-book, i.e., those covering health, stress, or anxiety, attracted scientists' attention more successfully by tackling people's most urgent problems head on. It is also plausible that sufficient data needed to address the under-represented issues were not available at the early stages of the pandemic. In any event, despite authors' preference for some topics over others, the less popular ones are no less important and comprise such hot topics as the relation between political attitudes and aspects of the pandemic or differences in moral decision making between frontline workers and lay people. To recap, despite a clear focus, the published articles deal with a wide range of timely and psychologically relevant problems triggered by the pandemic as illustrated by 465,389 views (up till July 21st, 2021). Moreover, a large majority-−82%—of the 65 articles report original research findings, while the remainder were written in a wide range of formats including seven brief reports, two conceptual analyses, one opinion and one perspective.

Another important aspect that helps us to interpret the keywords in Figure 1 is time. With time passing, we can observe changes in psychological and behavioral reactions to the pandemic. This is reflected in articles published in this e-book that could be divided into three groups: (1) dread and anxiety, (2) effects of social isolation and compliance with the lockdown, and (3) tiredness with restrictions and resistance. It is tempting to see these as resulting from three different phases of reactions to the pandemic.

## Dread and Anxiety

The call for submission appeared very early in the pandemic, and this came with advantages and disadvantages. At that time, a majority of people was terrified of the unknown disaster. Neither researchers nor the public had a hint of what would happen, how long this horror would last and what could be done to end the pandemic. Not surprisingly, in the manuscripts submitted early on, contributing authors attempted to generalize the experience from past pandemics to COVID-19. In this cognitively ambiguous and emotionally disturbing period, the first reaction of official agencies was to introduce lockdowns to stop the spread of COVID-19. This might have been motivated by the experience with Spanish flu and the story of two cities: Philadelphia and Saint Louis that reacted very differently to the 1918 flu pandemic. St. Louis was fast to act against the pandemic, whereas Philadelphia was not. In likely consequence the death rate was much higher in Philadelphia than in St. Louis (748 in comparison to 358 per 100,000 people). The lockdown, introduced in March 2020, initially enjoyed social support in most European countries and in the U.S., most likely because many people were concerned and considered COVID-19 to be an unknown risk. Therefore, in accordance with Slovic's taxonomy of risk (Slovic, 1987), the pandemic could be evaluated highly on both factors: dread and unknown risk.

## Effects of Social Isolation and Compliance With the Lockdown

From the early stages of the pandemic, psychologists have been strongly interested in the psychological reactions to lockdowns, such as perceived risk and emotions. These early but well justified concerns are well documented in this issue; many articles address pain of isolation, stress, and mental health in relation to personality traits, such as introversion, regulatory focus, anxiety and/or depression. Despite broad social support for the lockdown, the issue of variable compliance with lockdown restrictions was also studied from the very beginning of the pandemic, and one can find the articles examining this issue in this Research Topic.

With passing time, the focus shifted from psychological hardship and its consequences for mental health and well-being to the impact of lockdowns on cognitive processes and social behavior and adaptation to lockdowns were a focus. From these papers, one can learn about reading emotions from faces covered by facial masks, the value of alert systems and gentle reinforcement, effects on stigmatization, social trust, changes in consumption and trade-offs between public health and personal freedom. Some of these articles have received a great deal of attention; for example, one study investigating how the wearing of protective face masks confuses counterparts in reading the emotions of the mask wearer has had 61,922 views up until July 21st, 2021.

## Tiredness With Restrictions and Resistance

All indices of the pandemic went down during the summer 2020, which most likely resulted from the spring lockdowns. This and the fact that many people will have adapted to the threat might explain why support for more stringent measures to fight against the pandemic gradually waned over time. The slowdown in the pandemic and the adaptation to the threat combined with tiredness with restrictions and boredom during the vacation time to provoke some strongly voiced social protests against restrictions. These reactions were captured in manuscripts submitted during the summer of 2020 which could be seen as a third phase of pandemic reaction concerned more with resistance to and fatigue from pandemic constraints. Some of these papers focused on inappropriate public policies adopted to fight COVID-19 often arising from misinterpretation of statistical data (this is discussed in two conceptual analyses). In other manuscripts, compliance with preventive behavior was discussed in relation to risk perception, media communication and message framing, cognitive processes, and social and cultural factors. Finally, some manuscripts examined false beliefs and conspiracy theories.

## Current and Future Challenges

As we have already learned, the pandemic is a highly dynamic process. The call for proposal was closed in summer 2020 and so the changes in perception of the pandemic that happened subsequently are not captured in this e-book, with one exception of the research on post-pandemic consumption in China, where the restrictions were lifted earlier. In the immediate term, it is currently of great importance to monitor and understand attitudes toward the Covid-19 vaccination programs, which appear to change rapidly and involve both legitimate concerns and false beliefs. Vaccine hesitant behavior has encouraged some in public policy to look for effective reinforcements of pro-vaccine attitudes for those who are willing to get vaccinated to some restrictions for those who are not. For example, on May 12, 2021, the state of Ohio announced a lottery system to pay randomly selected vaccine recipients up to $1 million. After initial reports that vaccine uptake had subsequently increased in Ohio, other states adopted similar vaccine payment lotteries. Unfortunately, Walkey et al. (2021) did not find an increase in Ohio's vaccination rate in comparison with the rate in the U.S. As one might have expected both the rewards and the restrictions associated with vaccination are supported/opposed by different members of societies. Among vaccine policies the idea of a COVID vaccine passport seems to be one of the most controversial. Such controversies are interesting and perhaps somewhat surprising when one considers that vaccination certificates have been required for many years for foreign travel (e.g., the yellow fever vaccine for people who entered Seychelles) with no organized opposition from travelers. Similarly, most travelers accept security checks at airports, albeit often wearily, even though this represents an invasion of their privacy. This illustrates that personal and social reactions to limitations of personal freedom are guided by various cognitive and emotional processes as well as political and moral world views. Therefore, a good understanding of these factors is an important condition for delivering an unbiased and effective message to the public.

In a longer term perspective, the focus may shift to assessing the effectiveness of public policies adopted to fight Covid-19, and the long-term consequences of the pandemic for mental health, social relations, economics and educational system. For example, it would be interesting to investigate the extent to which changes in work habits, consumption and social relations forced by the pandemic will lead to permanent modifications of behavior. Crowded pubs, bars and restaurants are one example of an immediate response to lifting restrictions. In economics, this could be interpreted in terms of pent-up demand and accumulated savings (Sheth, 2020), which implies an optimistic vision of a returning to pre-pandemic levels of restricted behaviors. However, another possible explanation for the same effect might be given in terms of Brehm's (1966) theory of psychological reactance. In accordance with this theory, when behavioral freedom is reduced, individuals are motivated to regain it and then this could be a short time trend. Another open question is whether the switch to online purchases during the pandemic is likely to sustain even though most consumers feel safe to visit stores. Does such behavior become a habit or perceived as an involuntary choice? The same question could be asked about virtual social communication, online teaching and working from home. Which hypotheses are more accurate is of great importance for the prediction of post-pandemic market behavior.

## Conclusion

Even more generally, the pandemic can be viewed as an extraordinary, global, “natural” experiment that may bring, among others, a better understanding of issues that have been studied for many years, such as the conflict between personal freedom and compliance with social policies unfriendly to such freedoms, or factors affecting economic behavior, as well as enhanced awareness of both advantages and limitations of new phenomena, such as virtual communication between people.

Along with many other special issues of journals our Research Topic has published a large number of studies addressing a wide range of topics relevant for understanding the human behavioral response to the Covid-19 pandemic. The pandemic continues to pose a threat and continues to provide new challenges and opportunities for psychological science; given the large global numbers of unvaccinated people, and the potential for new, more infectious and more lethal variants, it is not difficult to imagine the continuation of the research challenge for some time to come. In any event people seeking to manage future pandemics, and indeed other human crises requiring the prediction and understanding of human behavior, may draw on the research presented here.

## Author Contributions

All authors listed have made a substantial, direct and intellectual contribution to the work, and approved it for publication.

## Conflict of Interest

The authors declare that the research was conducted in the absence of any commercial or financial relationships that could be construed as a potential conflict of interest.

## Publisher's Note

All claims expressed in this article are solely those of the authors and do not necessarily represent those of their affiliated organizations, or those of the publisher, the editors and the reviewers. Any product that may be evaluated in this article, or claim that may be made by its manufacturer, is not guaranteed or endorsed by the publisher.
